# Supplementary material for: Detecting the outbreak of influenza based on the shortest path of dynamic city network
Source: PeerJ. 2020 Jul 20;8:e9432. doi: 10.7717/peerj.9432 (PMC7377247; doi:10.7717/peerj.9432)
Supplement: Supplemental Information 1 [file peerj-08-9432-s001.docx]

**Supplementary Information of Detecting the outbreak of influenza based on the shortest path of dynamic city network**

**Supplementary Information**


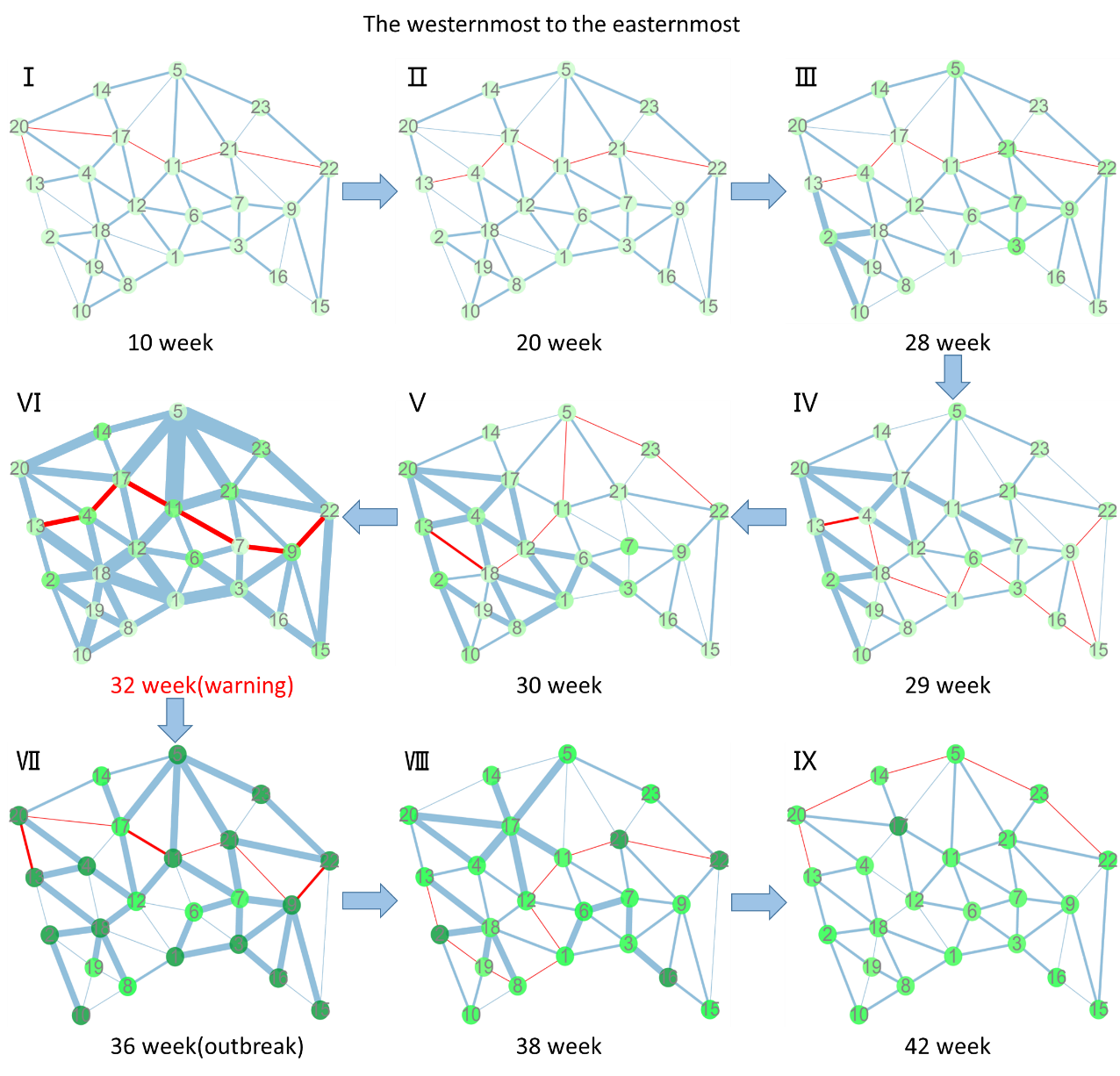


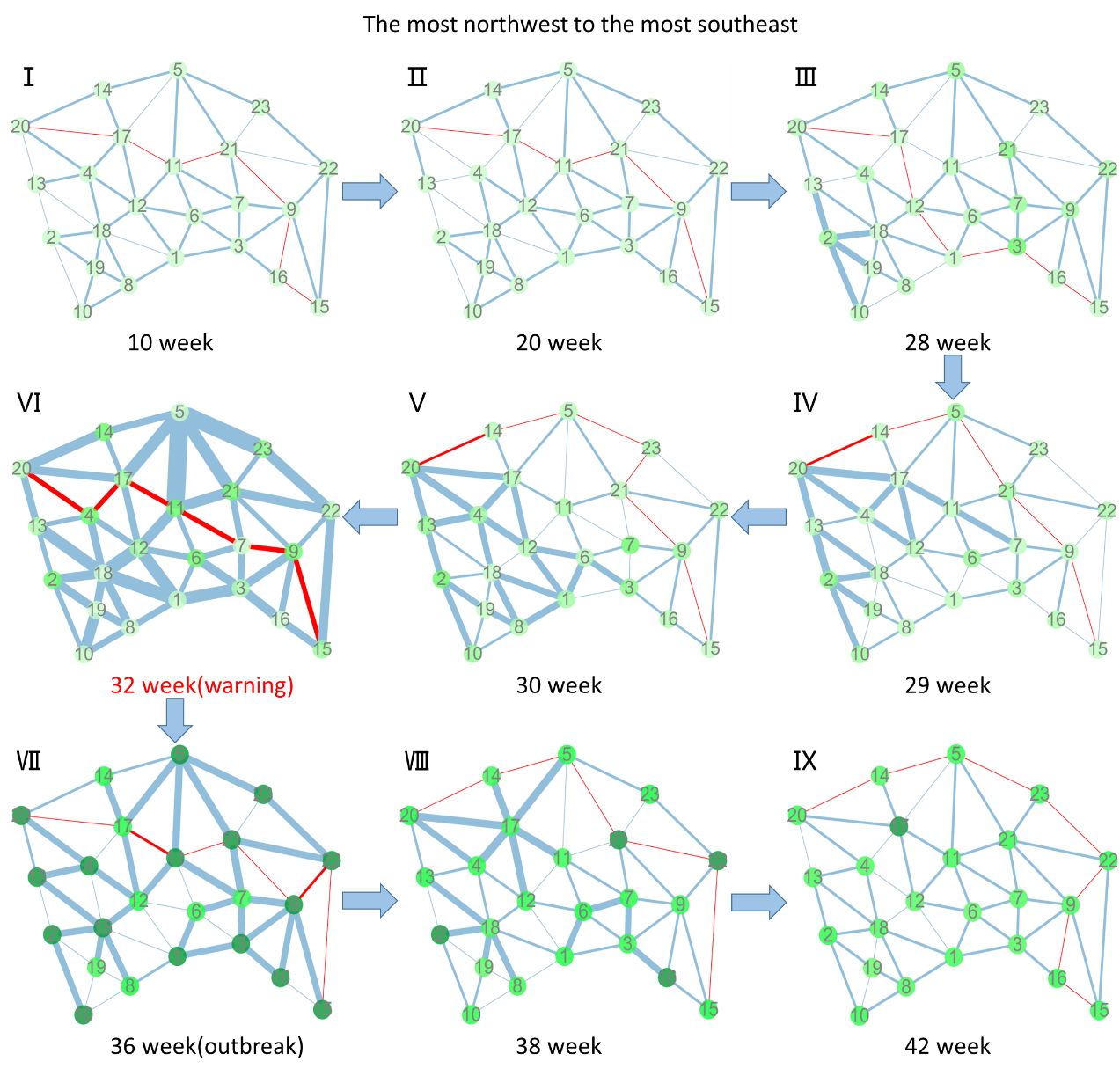


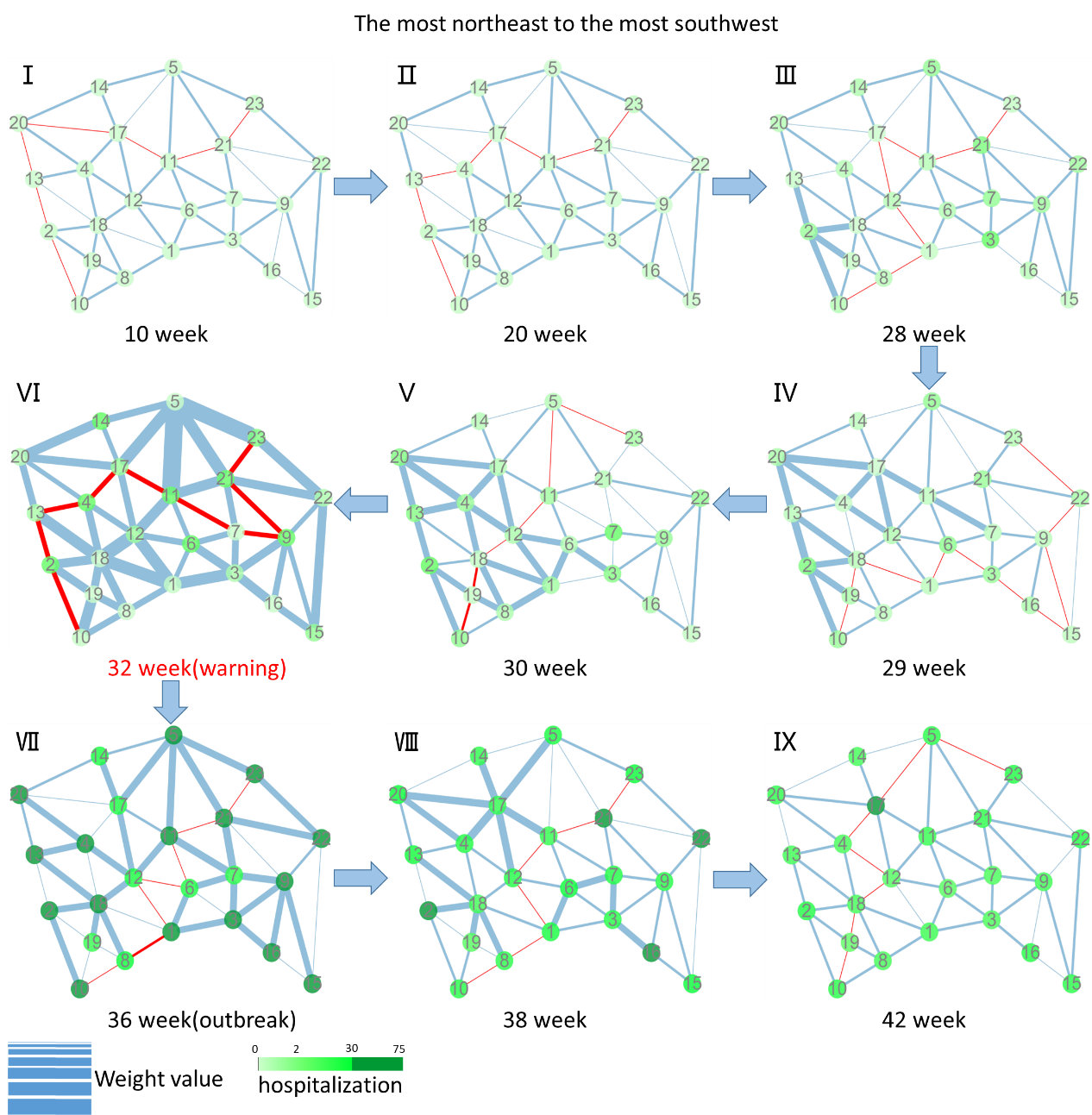


**Figure S1: The Dynamic evolution of Tokyo city network during the key period with the other three shortest paths from 2013 to 2014.** The thickness of edges represents the weight value. And path in red shows the shortest path from the northernmost node to the southernmost node. Nodes are colored by the number of clinic-visiting count in corresponding region. Including the westernmost to the easternmost, the most northwest to the most southeast and the most northeast to the most southwest, It is clear that the shortest path is stable in the normal state (the 10th-28th weeks), while a drastic change appears at the 32th week just before the influenza outbreak.


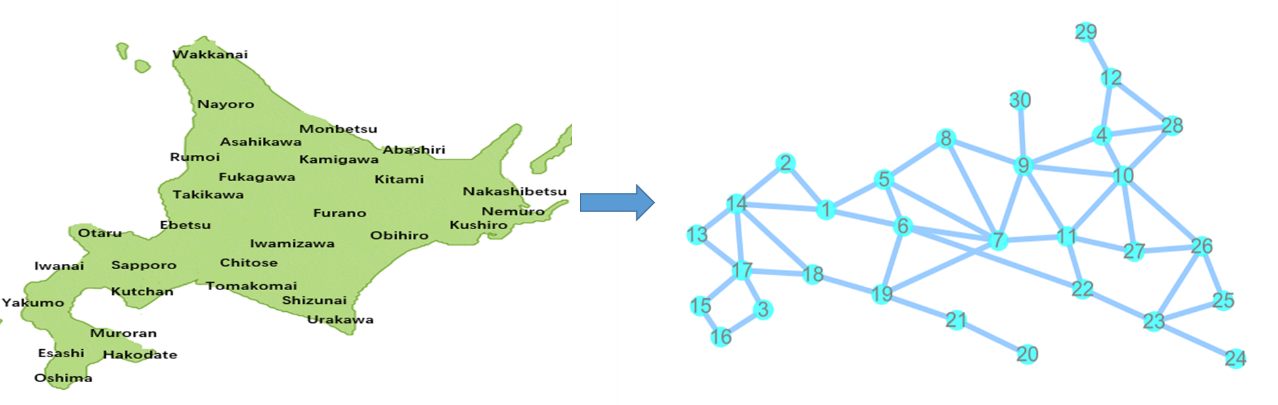


**Figure S2: Original map of Hokkaido and its constructed network.** In Hokkaido, four shortest paths we defined are: (1) node 30, Rumoi to node 20, Urakawa, represents northernmost node to southernmost node; (2) node 13, Iwanai to node 26, Abashiri, represents westernmost node to easternmost node; (3) node 2, Otaru to node 24, Nemuro, represents the most northwest node to the most southeast node; (4) node 16, Oshima, to node 29, Wakkanai, represents the most northeast node to the most southwest node.


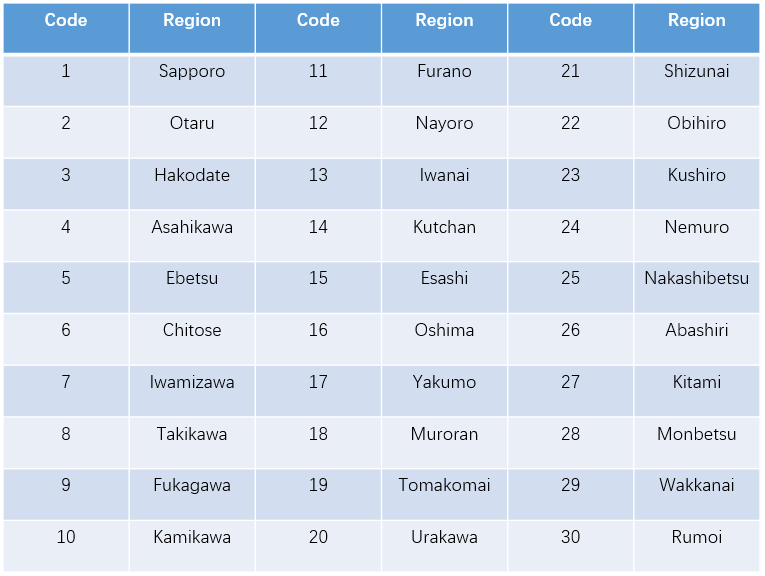


**Table S1: The name of 30 regions in Hokkaido and its corresponding code**


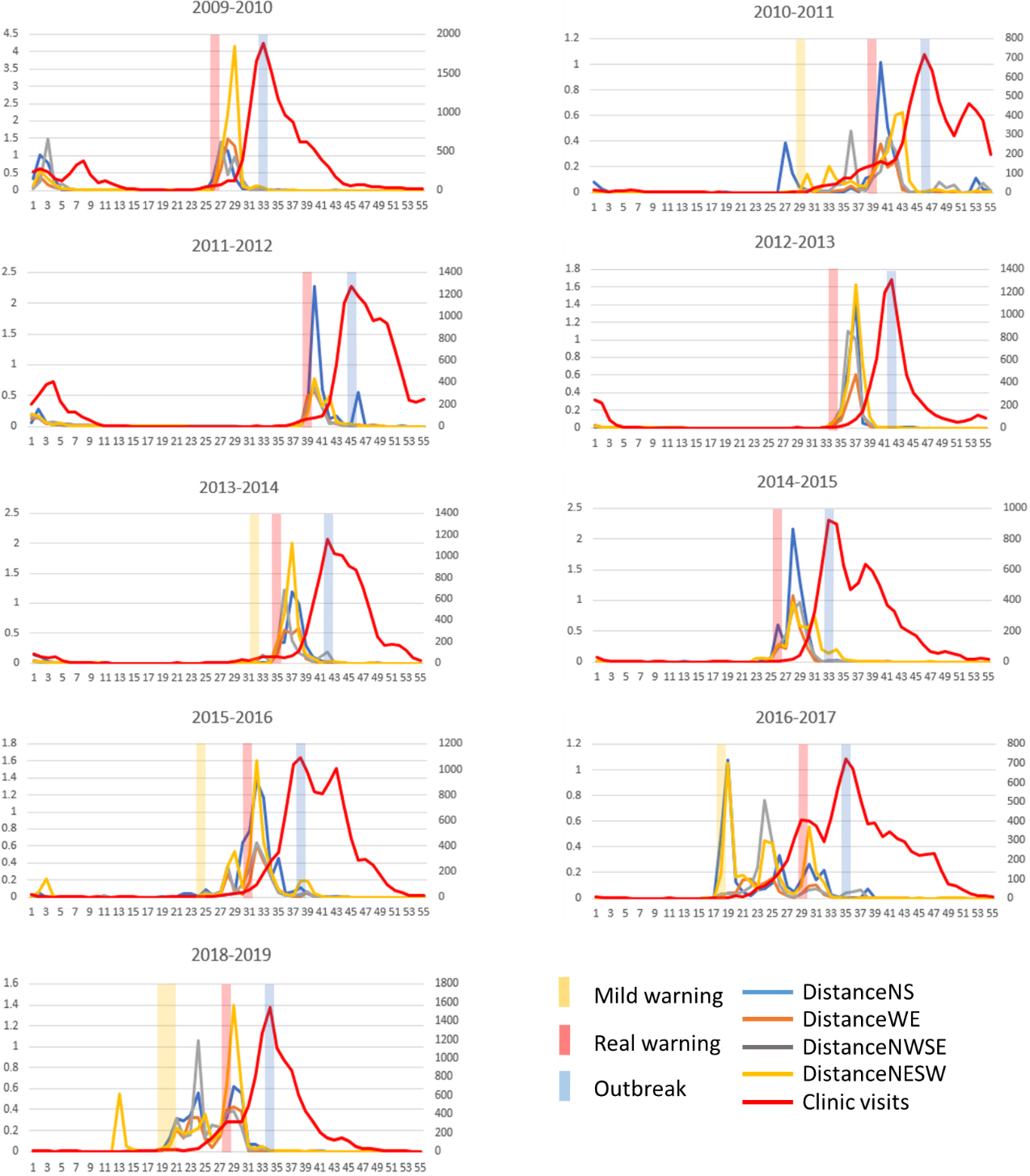


**Figure S3: The predictions of annual influenza outbreak in Hokkaido from 2010 to 2019.** For each year, our SP-DNM method timely detect the early-warning signal of influenza outbreak. The red line represents the clinic-visiting count, while the other lines represent the SP-DNM scores. X axis represents the time evolution in a year. Y axis in left side represents SP-DNM scores, and Y axis in right side represents the clinic-visiting count. The yellow bar stands for mild warning, red bar represents real warning and blue bar represents the influenza outbreak point.


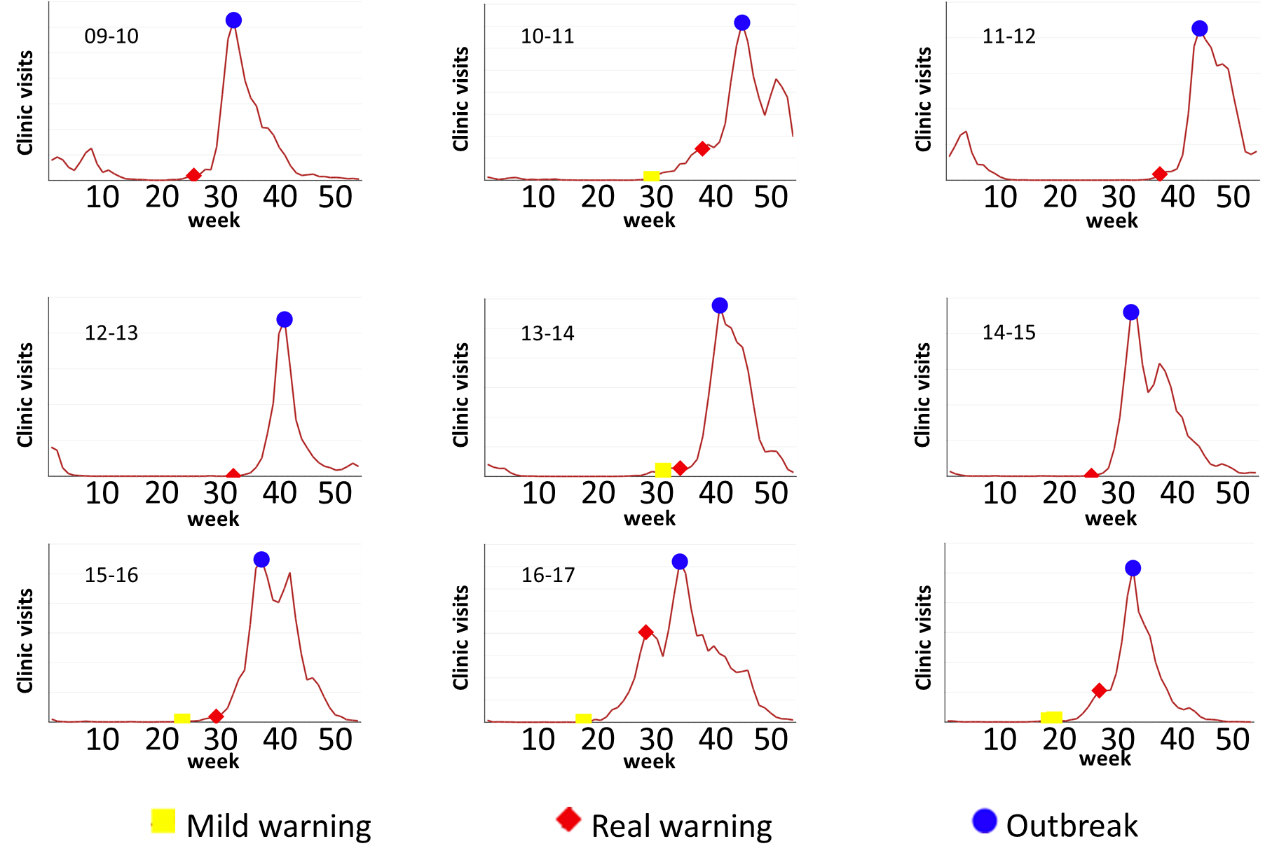


**Figure S4: Identified results by SP-DNM in Hokkaido from 2010-2019.** It is clear that flu outbreak was accurately detected by SP-DNM method. The Y axis represents the number of clinic visits, yellow rectangle represents the mild warning point, red diamond represents real warning point, blue circle represents flu outbreak point.


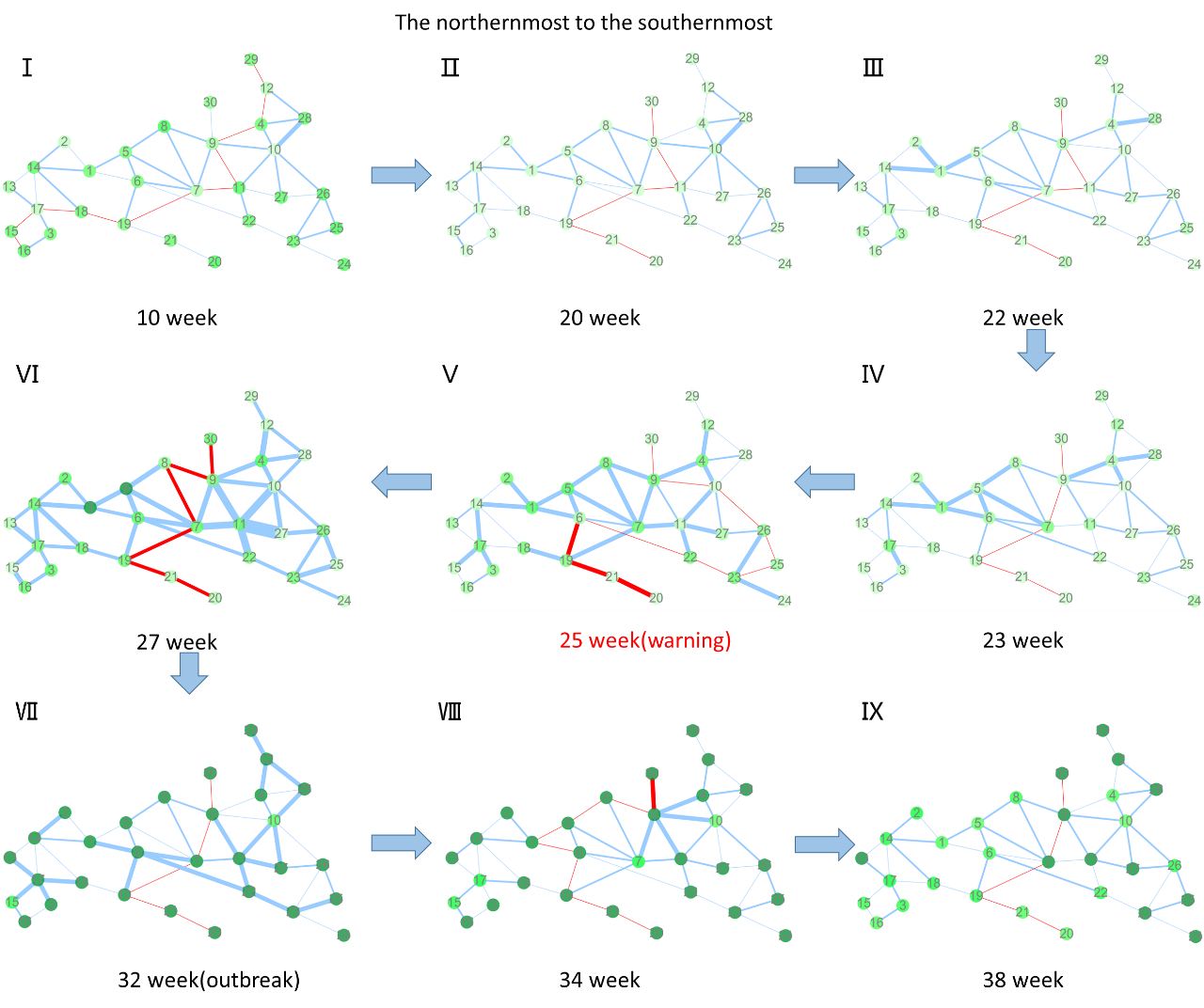


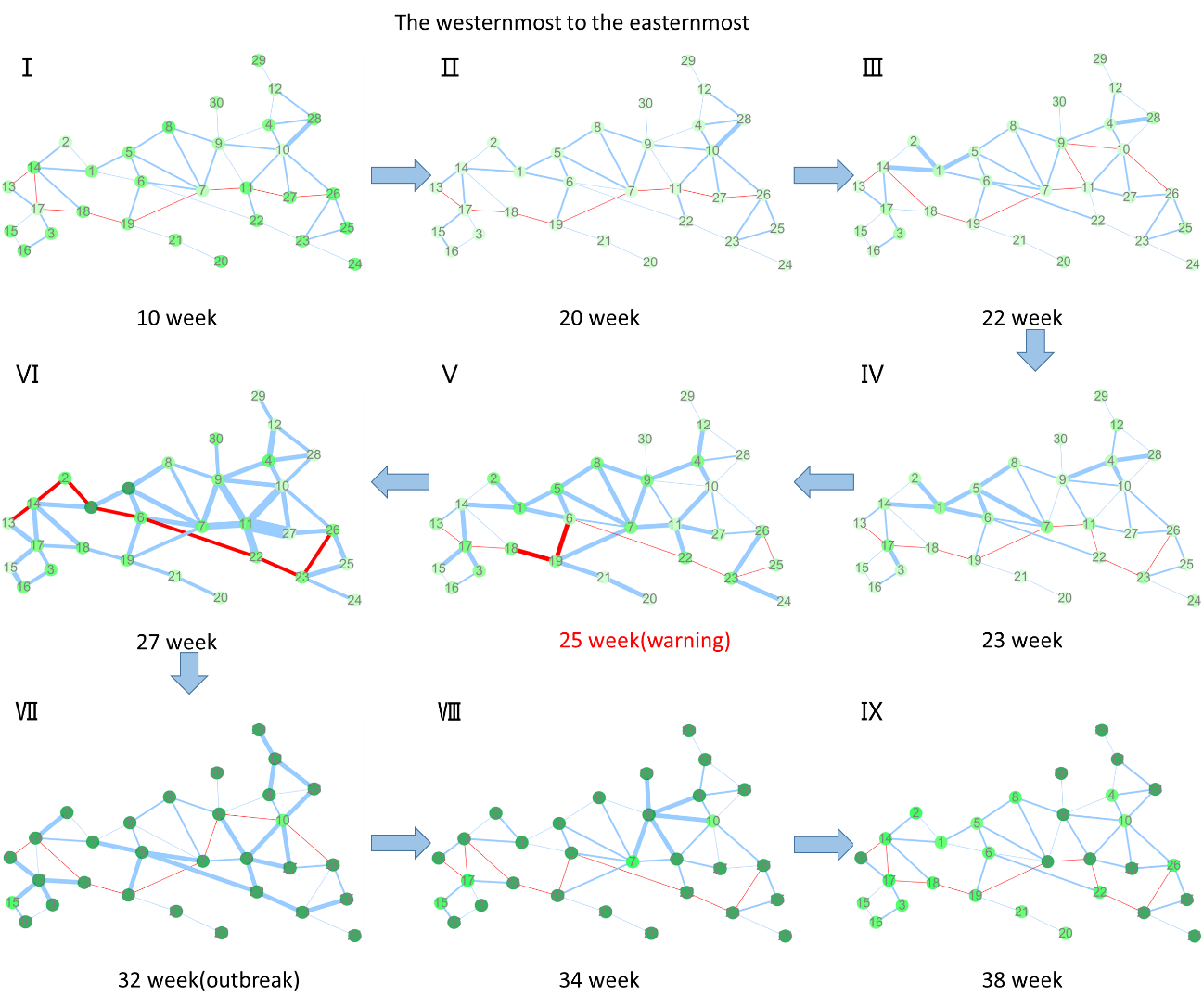


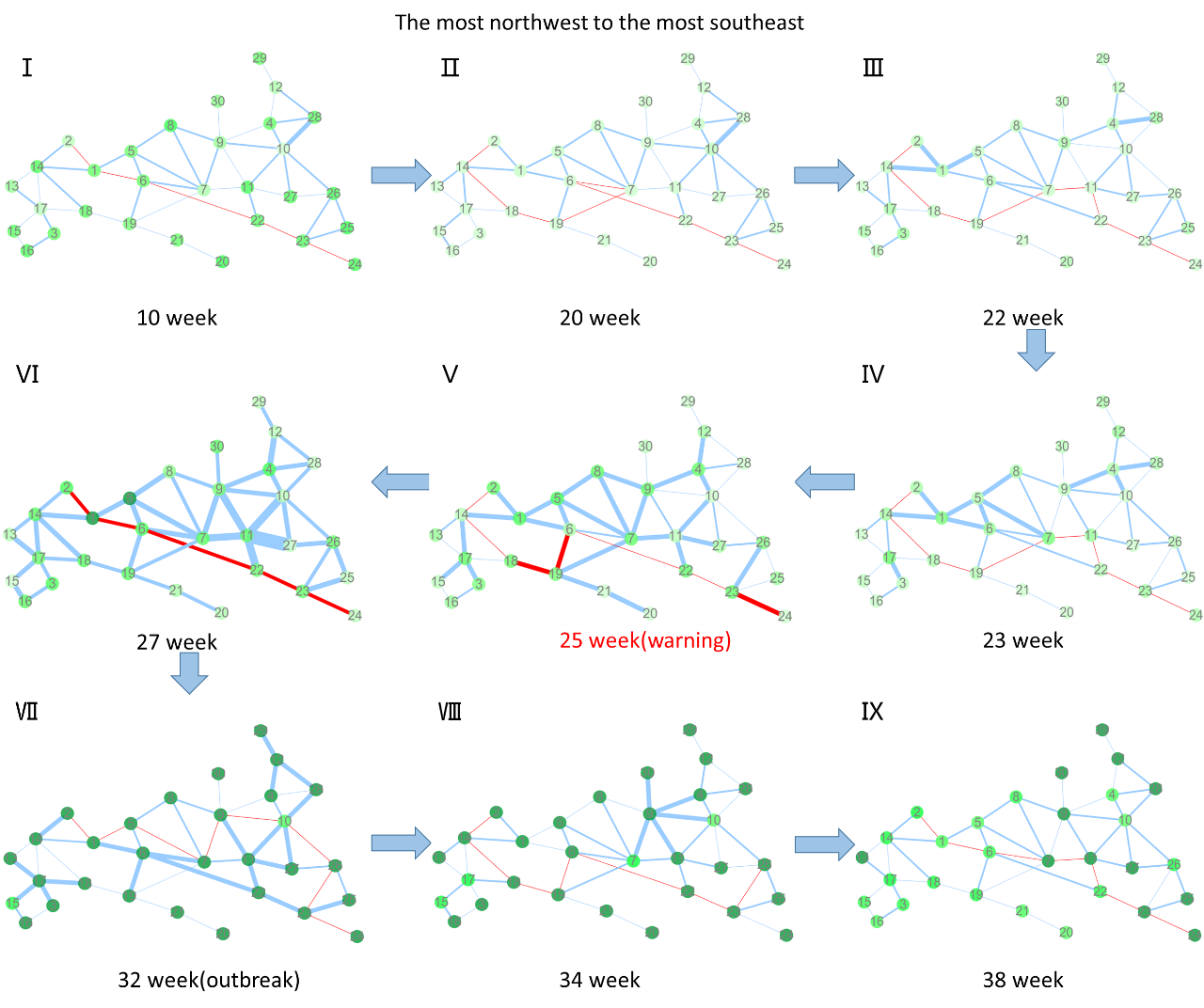


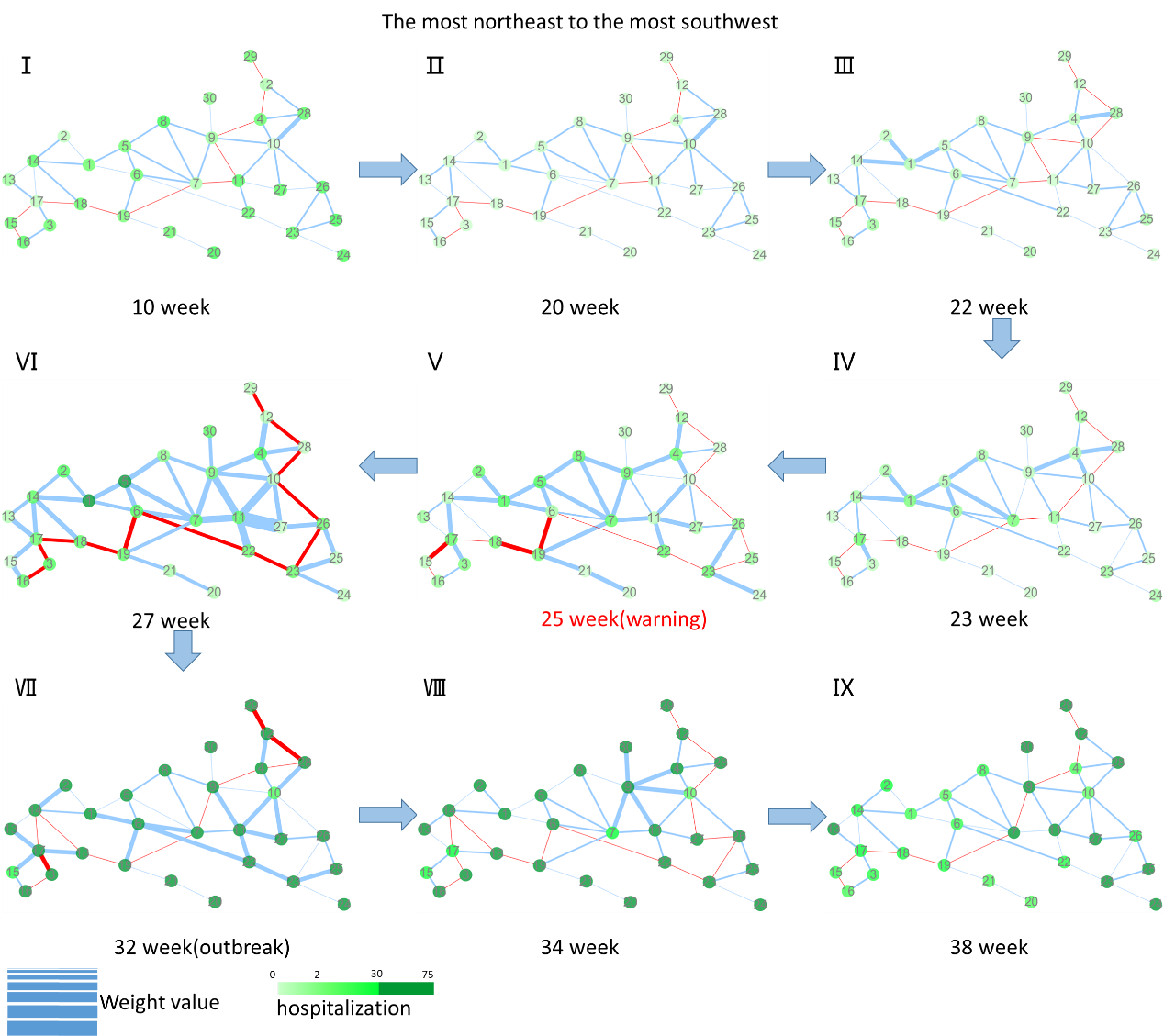


**Figure S5: The dynamic evolution of Hokkaido city network during a key period from 2009 to 2010.** The thickness of the edges represents the correlations between corresponding nodes (calculation in second step of algorithm). The thickness of edges represents the weight value. And path in red shows the shortest path in the map. Nodes are colored by the number of clinic-visiting count in corresponding region. It is clear that the shortest path is stable in the normal state (the 10th-20th weeks), while changes firstly occurred near node 1 and node 4 at 22^th^ week. And as time goes by, the influenza impact gradually spread to farther region, leading more and more edges near node 1 and node 4 become thicker. At 25^th^ week, both standard deviation and correlation between adjacent node increase sharply, generating early warning signal before flu outbreak at 32^th^ week.
